# Interocular suppression in chromoluminance patterns measured with SSVEP

**DOI:** 10.1167/jov.25.4.6

**Published:** 2025-04-15

**Authors:** Alex A. Carter, Daniel H. Baker, Antony B. Morland, Abbie J. Lawton, Alex R. Wade

**Affiliations:** 1 Department of Psychology, University of York, York, UK; 2 York Biomedical Research Institute, University of York, York, UK

**Keywords:** color, binocular, SSVEP, normalization

## Abstract

Many cells in the early human visual system respond to either chromatic or luminance contrast or a combination of both. In addition, depending on their location in the visual hierarchy, these cells may receive input from either one eye or both eyes. It is well understood that spatial luminance contrast patterns undergo binocular normalization: Inputs from each eye mutually suppress each other so that monocular and binocular percepts appear similar. Recent reports suggest that interocular normalization computations may depend on spatial and temporal frequency. Here, we examined the effect of chromaticity and spatial frequency on binocular normalization computations using a dichoptic frequency-tagged, steady-state visually evoked potential (SSVEP) paradigm. We found that normalization as indexed by changes in eye-tagged input SSVEP frequencies and intermodulation terms depends significantly on both spatial frequency and color. We also found that binocular combination must occur in neurons that carry half-wave rectified signals due to 1*F* combination frequencies being present. Overall, our results are not well explained by a model in which neurons that code low spatial frequency color are segregated anatomically in the centers of ocular dominance columns. Significant levels of binocular interaction must occur in neurons that code both color and luminance and in neurons sensitive to both low and high spatial frequencies.

## Introduction

The world does not get dimmer when we close one eye. This observation has driven over a century of research into the rules governing binocular combination: the way that the visual system combines inputs from our two eyes. Many contemporary models propose that monocular signals are combined following a normalization stage where a suppressive drive is provided by the other eye (Baker & Wade, 2017; Ding & Sperling, 2006; Hou, Nicholas, & Verghese, 2020; Meese, Georgeson, & Baker, 2006; Moradi & Heeger, 2009; Segala, Bruno, Martin, Aung, Wade, & Baker, 2023). This normalization must happen at a point in the visual system that contains binocularly responsive cells, and most models assume that this occurs in V1. Neurons prior to V1 (for example, in the lateral geniculate nucleus) receive monocular bottom-up input, and almost all neurons after V1 are binocularly responsive. V1 itself contains a wide range of cells with different levels of ocular dominance (Hubel & Wiesel, 1962): On average, most cells have some degree of binocularity but a significant fraction, particularly in the input layers, inherits specificity for a single eye.

Cells are spatially organized according to their preferred stimulus features in primate visual cortex. Cytoarchitectural studies in macaques indicate the presence of spatial maps for chromatic tuning, binocularity, orientation preference, and spatial frequency (Adams & Horton, 2009; Chatterjee, Ohki, & Reid, 2021; Johnson, Hawken, & Shapley, 2008; Livingstone & Hubel, 1988; Nauhaus, Nielsen, Disney, & Callaway, 2012). These spatial maps are aligned to some extent; for example, there is evidence that the centers of chromatic tuning maps are coincident with the locations of cytochrome oxidase “blobs” (Chatterjee et al., 2021; De Valois & Pease, 1971; Edwards, Purpura, & Kaplan, 1995; Garg, Li, Rashid, & Callaway, 2019; Li, Garg, Zhang, Rashid, & Callaway, 2022; Thorell, de Valois, & Albrecht, 1984). In turn, these may align with the centers of ocular dominance columns (Livingstone & Hubel, 1988), and spatial frequency tuning (as well as binocularity) may change as a function of the distance of a cell from the center of cytochrome oxidase blobs. This suggests that the level of interocular normalization that a binocularly presented stimulus experiences might be a function of all three stimulus properties.

However, there is some reason to re-examine these assumptions about co-varying stimulus properties. First, the classifications are not binary; for example, macaque V1 contains many chromatically responsive cells that sit outside the cytochrome oxidase blobs (Chatterjee et al., 2021; Garg et al., 2019; Lennie, Krauskopf, & Sclar, 1990; Leventhal, Thompson, Liu, Zhou, & Ault, 1995; Li et al., 2022), the degree of orientation tuning inside the cytochrome oxidase blobs is not significantly different from that found outside (Economides, Sincich, Adams, & Horton, 2011; Leventhal et al., 1995), significant levels of functional signal mixing occur between V1 inputs and outputs (Sincich & Horton, 2005), and not all members of a primate species necessarily have the same columnar organization of ocular dominance preferences (Adams & Horton, 2009). Moreover, almost all of our data regarding the ocularity of V1 neurons comes from animal models (cats and non-human primates). Measuring the structure of ocular dominance columns in humans is notoriously difficult even with high-field imaging systems (Cheng, Waggoner, & Tanaka, 2001; de Hollander, van der Zwaag, Qian, Zhang, & Knapen, 2021; Nasr et al., 2024; Yacoub, Shmuel, Logothetis, & Uğurbil, 2007).

Therefore, it is not possible to make strict binary functional distinctions between, for example, different chromatic pathways or pathways originating in specific retinal cell types (e.g., magnocellular and parvocellular cells) (Levitt, Schumer, Sherman, Spear, & Movshon, 2001), especially after the first synapse (Sincich & Horton, 2005). Some support for stimulus dependence has come from recent work suggesting that interocular normalization may be weaker when achromatic input stimuli have low spatial and low temporal frequencies (Segala et al., 2023) compared with previous work with high spatial frequency stimuli. Here, we extend this work to examine the effect of changing both the color (defined in MacLeod–Boynton cone contrast space) (MacLeod & Boynton, 1979) and spatial frequency.

The primate visual system contains a variety of cell types, including those responding primarily to signed changes in color or luminance and those responding to the presence of spatial edges in a relatively phase insensitive manner. The nomenclature for these cells can sometimes depend on the stimulus used to examine them: In the framework of achromatic contrast signaling, simple cells typically respond to signed excursions (light or dark) but complex cells respond to spatial edges in a phase insensitive manner (Hubel & Wiesel, 1962). Although this apparently bimodal segregation into simple and complex cell types can arise from a nonlinearity in the response stages of cells with a far more uniform distribution of receptive field properties (Mechler & Ringach, 2002), we maintain the terminology here as a convenient way of classifying cells by their contribution to different response frequency components. In the domain of color vision and opponent-color theory, a similar distinction is often made between single opponent cells (responding to a signed excursion in cone contrast space with effectively no spatial discrimination) and double opponent cells that can signal spatially structured chromatic patterns ranging from center/surround organizations similar to achromatic simple cells through to phase-insensitive edge detection and chromatic borders depending on the receptive field structure (e.g., Johnson et al., 2008). Here, we use a simplified terminology to refer to cells that signal a signed excursion in three-dimensional (3D) chromatic space as “simple” cells and those that signal edges (either chromatic or achromatic) in a phase insensitive manner as “complex” cells (e.g., Solomon, Peirce, & Lennie, 2004). We note that many cells in primary visual cortex that are “simple” for achromatic contrast (e.g., responding to signed luminance inputs with center/surround structure) are also “simple” under this definition for chromatic contrast because the cone inputs to the center and surround of the receptive field are rarely sufficiently balanced to achieve perfectly null responses to L–M and S–(L+M) that cover the entire receptive field.

For a contrast-reversing flicker at frequency *F*, ideal simple cells respond at the fundamental frequency (1*F*), reflecting their sensitivity to polarity-specific input, whereas ideal complex cells respond at the second harmonic (2*F*), reflecting their sensitivity to the temporal changes in contrast polarity, which occur twice per cycle. A useful way to probe visual responses is to use a steady-state visually evoked potential (SSVEP) paradigm, in which flickering stimuli entrain cortical responses at harmonics of their flicker frequencies (Norcia, Appelbaum, Ales, Cottereau, & Rossion, 2015), which are measured using electroencephalography (EEG). In the SSVEP, nonlinear binocular combination manifests in two ways: (a) suppression between the signals in the left and right eyes, measured by a reduction in the input frequencies (self terms); and (b) the generation of novel intermodulation (IM) terms: sums and differences of self-term harmonics. Here, we assess how these responses change across chromaticity and spatial frequency, and we assess differences in how binocular combination affects responses of signals due to simple and complex cells.

## Methods

### Experiment 1

#### Participants

Twelve participants (four male, eight female; mean age ± *SD*, 21.6 ± 2.51 years) took part in the study. All participants had normal or corrected-to-normal vision including normal color vision as assessed by pseudoisochromatic plates and psychophysical isoluminance settings. Informed consent was obtained from each participant, and procedures were approved by the ethics committee of the Department of Psychology at the University of York. Procedures adhered to the tenets of the Declaration of Helsinki.

#### Apparatus and stimuli

**Figure 1.**
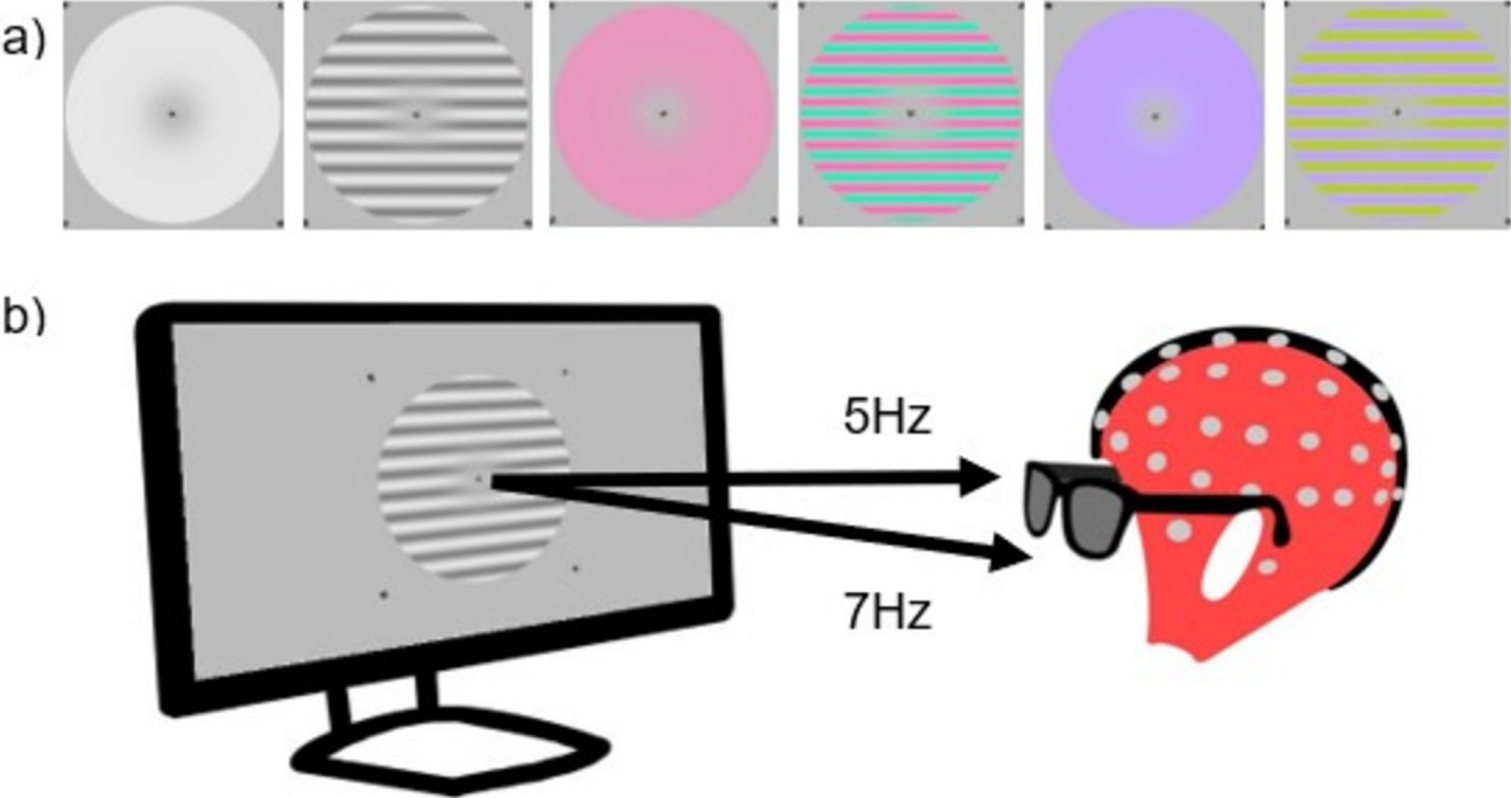
(**a**) Examples of stimuli used in each condition: luminance, L-M, and S-cone for disks and gratings, respectively. (**b**) Example setup with the same stimulus presented to each eye at different frequencies using the shutter goggles.

The experimental display system is shown in Figure 1b. Stimuli were presented on a gamma-corrected VIEWPixx/3D LCD monitor (VPixx Technologies, Saint-Bruno, QC, Canada) with a resolution of 1920 × 1080 pixels and refresh rate of 120 Hz. Eyes were targeted using stereo shutter goggles (3D Vision Pro 2; NVIDIA, Santa Clara, CA) which were synchronized with the refresh rate of the monitor via an infrared connection. EEG data were collected using a waveguard 64-channel cap (ANT Neuro, Blackburn, Victoria, Australia) and ANT Neuro EEG system at a sample rate of 1000 Hz. Triggers from the stimulus computer indicating trial onset and condition were transmitted to the EEG amplifier via an 8-bit parallel port. Participants were seated 57 cm from the stimulus display.

Stimuli consisted of contrast-reversing flicker at 5 Hz into the right eye only, 7 Hz into the left eye only, or 5 Hz into the right eye and 7 Hz into the left eye simultaneously (three ocularity conditions). A photodiode (SM1PD1B; Thorlabs, Newton, NJ) attached to an oscilloscope (Picoscope 2204A; Pico Technology, St Neots, UK) was used to ensure that flicker frequencies were accurate, that the rise and fall times on the monitor were close to those quoted by the manufacturer (1 ms), and that the stimulus input waveforms were sinusoidal and therefore generated little or no harmonic distortion at the input stage. Stimuli were either disks or horizontal sine-wave gratings with a spatial frequency of 0.5 c/deg that targeted the three post-retinal pathways (luminance, L-M, or S-cone). The relatively low grating spatial frequency was chosen to ensure that a single pattern drove both chromatic and achromatic receptive fields in primary visual cortex in the eccentricity range of 2° to 10°. Chromatic and achromatic stimuli with spatial frequencies in this range elicit robust and relatively similar population responses in primary visual cortex (Welbourne, Morland, & Wade, 2018). Figure 1a shows examples of the stimuli used. There were 18 conditions: six stimulus conditions factorially combined with three ocularity conditions. Contrasts were 30%, 60%, and 70% of maximum cone contrast that our system could display for luminance, L-M, and S-cone stimuli, respectively (root mean square [RMS] cone contrasts of 0.300, 0.036, and 0.420, respectively).

Stimuli were displayed on a background of mean luminance of 33 cd/m^2^ (with goggles) and subtended 20 degrees of visual angle. The center of the stimuli was blanked out using a 2°-radius disk with raised cosine edges. Stimuli were computed in Macleod–Boynton color space and converted to RGB values using spectral calibrations taken through individual left and right shutter goggles with a JAZ Spectrometer (Ocean Optics, Orlando, FL) and the Stockman 10° cone fundamentals (Stockman & Sharpe, 2000). A fixation point was present in the center throughout, which was a small pattern made from squares both randomly positioned and a random shade of gray. This fixation pattern could change on each trial with a probability of 50%. To maintain attentional state, participants were required to maintain focus on the fixation point and click the mouse each time it changed. Isoluminant points were measured for each participant, and stimuli were corrected before the experiment began.

Trials consisted of 12 seconds of flicker with a 3-second interstimulus interval; we deliberately avoided longer blocks to minimize the effects of contrast adaptation (Webster & Mollon, 1991; Zhang, Valsecchi, Gegenfurtner, & Chen, 2023). There were 18 trials per block: one of each condition, presented in a randomized order. Participants completed eight blocks (each lasting approximately 4.5 minutes), with breaks after every two blocks.

#### Data analysis

EEG data for each participant were analyzed in MATLAB (MathWorks, Natick, MA). The initial 2 seconds of data from each trial were removed to eliminate onset transients. A bandpass filter was applied from 1 Hz to 35 Hz. Little noise rejection other than bad channel rejection and exclusion of very noisy bins (bins whose variance, or power, was more than 3 *SD* above the mean) was performed (Delorme, 2023). An electrode template for V1 was then applied to our data (Poncet & Ales, 2023). This template weights the electrodes based on the proportion of their signal being produced by left and right hemisphere V1.

Data were separated into 1-second bins and averaged within trials. The phases of responses for repeats of the same condition within hemisphere and subject were highly consistent. However, the phases of the data across participants and across left and right V1 within a participant were not consistent. So we averaged per-subject data coherently within hemispheres, and we averaged data across subjects and hemispheres incoherently by taking the scalar amplitude of the Fourier response. Amplitudes were converted to signal-to-noise ratios (SNRs) by dividing each frequency response by the RMS amplitude of all non-signal frequencies between 1 and 35 Hz. To allow further comparisons across key frequencies, we then fitted and removed a 1/*f* curve using the MATLAB lsqcurvefit function.

#### Statistical analysis

Statistical analyses were performed in MATLAB, Python, and Jasp (JASP Team, 2024).

### Experiment 2: control

Data from Experiment 1 were based on a stimulus configuration where the left eye input modulated at 7 Hz and the right eye at 5 Hz. To control for possible systematic eye dominance effects, we also ran a smaller set of control experiments where the eye frequencies were reversed. Five participants from the original sample (two male, three female; mean age ± *SD*, 26.8 ± 8.11 years) repeated the experiment with the frequency tags switched (5 Hz in left eye and 7 Hz in right eye); otherwise, the set-up and analyses remained the same.

## Results

### Experiment 1

**Figure 2.**
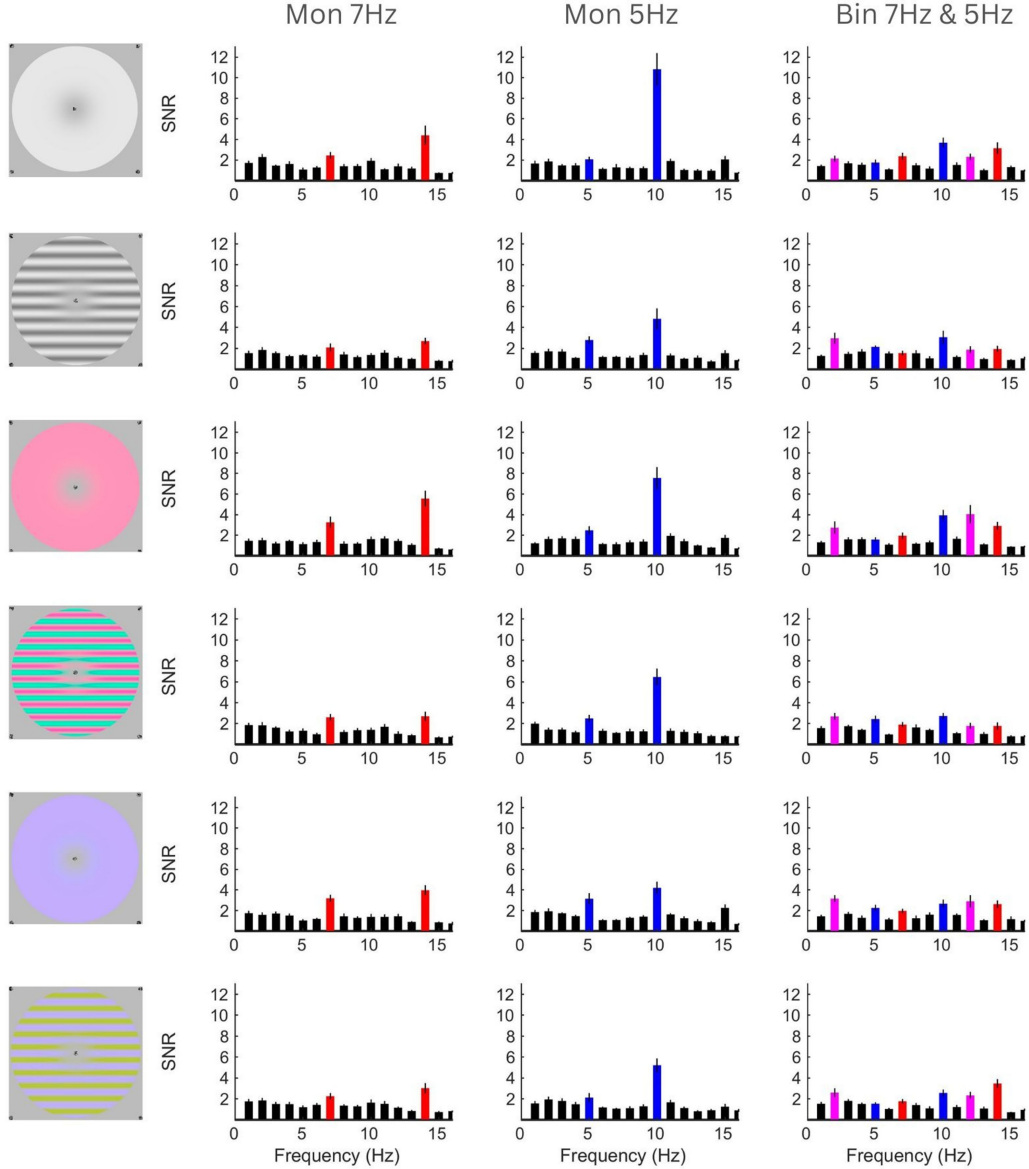
Signal-to-noise ratio of EEG data for each condition. Columns represent left monocular 7 Hz, right monocular 5 Hz, and binocular conditions, respectively. Rows represent the different stimulus conditions. Red, blue, and magenta bars highlight responses dependent on 7-Hz input, 5-Hz input, or binocular interaction, respectively. Error bars indicate 1 *SE*.

Figure 2 shows the SNR data for each condition. Columns show responses to 7-Hz flicker in the left eye only, 5-Hz flicker in the right eye only, and binocular flicker (7 Hz in the left eye, 5 Hz in the right eye). In monocular conditions, we see 2*F* responses and some 1*F* responses. We see these same responses in binocular conditions at a reduced amplitude, with additional responses at sums and differences of the 1*F* terms.

### Suppression

Suppression is the reduction of response in binocular conditions compared with monocular conditions. In 7-Hz monocular conditions, paired-samples *t*-tests revealed that significant self-terms were present compared with noise at both 7 Hz and 14 Hz (Figure 2, left column; *p* ≤ 0.003 and *p* ≤ 0.003, respectively). In 5-Hz monocular conditions, there were also significant self-terms at 5 Hz and 10 Hz (Figure 2, middle column; *p* ≤ 0.010 and *p* ≤ 0.002, respectively). In binocular conditions, these self-term responses remained significant compared with noise (Figure 2, right column; 7-Hz *p* ≤ 0.005, 14-Hz *p* ≤ 0.014, 5-Hz *p* ≤ 0.006, and 10-Hz *p* ≤ 0.004), but 2*F* responses were generally (in 10/12 cases; on average *p* ≤ 0.022) significantly reduced compared with the corresponding monocular condition in a paired-samples *t*-test.

**Figure 3.**
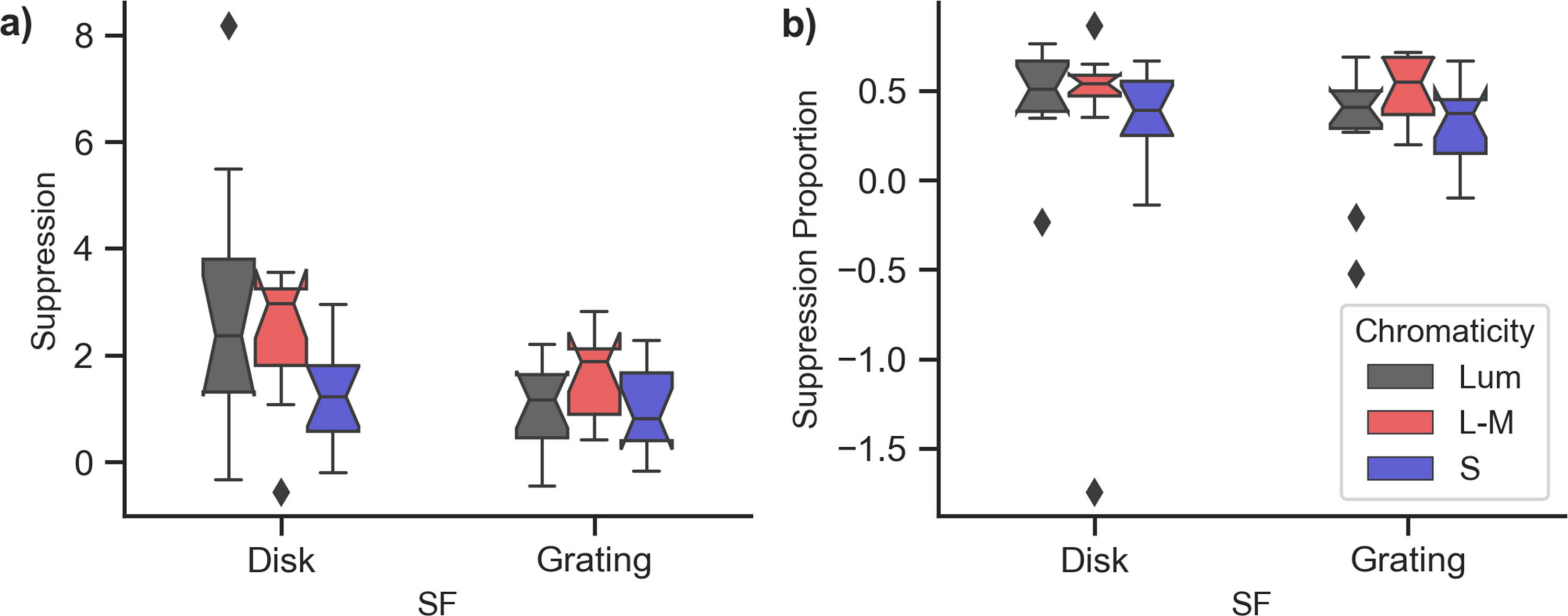
Boxplots of the average suppression at 1*F*1, 1*F*2, 2*F*1, and 2*F*2 by chromaticity and spatial frequency. Notches show 95% confidence intervals. (**a**) Change in raw amplitude (*mon* – *bin*). (**b**) Change in amplitude as a proportion of the monocular response ($\frac{mon-bin}{mon}$ ). When expressed as proportions of the monocular response, there was no significant effect of chromaticity or spatial frequency on normalization level.

Figure 3a shows boxplots of the average suppression of power at 1*F* and 2*F* self-terms across both eyes for each stimulus condition. An analysis of variance (ANOVA) on suppression values (*mon* – *bin*) found significant effects of chromaticity (*p* = 0.002) and spatial frequency (*p* = 0.005), as well as a significant interaction (*p* = 0.020). However, when suppression was normalized by the monocular response ($\frac{mon-bin}{mon}$), we found a very different pattern (Figure 3b). Here, an ANOVA on the normalized suppression values found no significant effects of chromaticity (*p* = 0.823) or spatial frequency (*p* = 0.560), and no significant interaction (*p* = 0.059). We expect the visual system to be more sensitive to proportional changes rather than absolute changes in response. So, this proportional change is more likely to be representative of perception.

### ON/OFF asymmetry

For a contrast-reversing full-field input, simple cells will respond to a single input polarity and therefore respond at 1*F*. However, because EEG measures a population response and because the visual system contains cells responding to both stimulus polarities, the measured SSVEP signal will not contain 1*F* components unless the ON and OFF populations are unbalanced in some way. Second-harmonic (2F) responses will therefore be produced by both the overall sum of a balanced simple cell population and by complex cells that will respond to the changes in polarity in the same stimulus. The degree to which simple cell responses are not balanced between the two stimulus polarities will be reflected in the relative amount of first-harmonic responses in the disk conditions and may reflect differences in ON and OFF simple cell population sizes, their response dynamics, or their ability to generate a far-field electrical potential that can be detected at the scalp.

**Figure 4.**
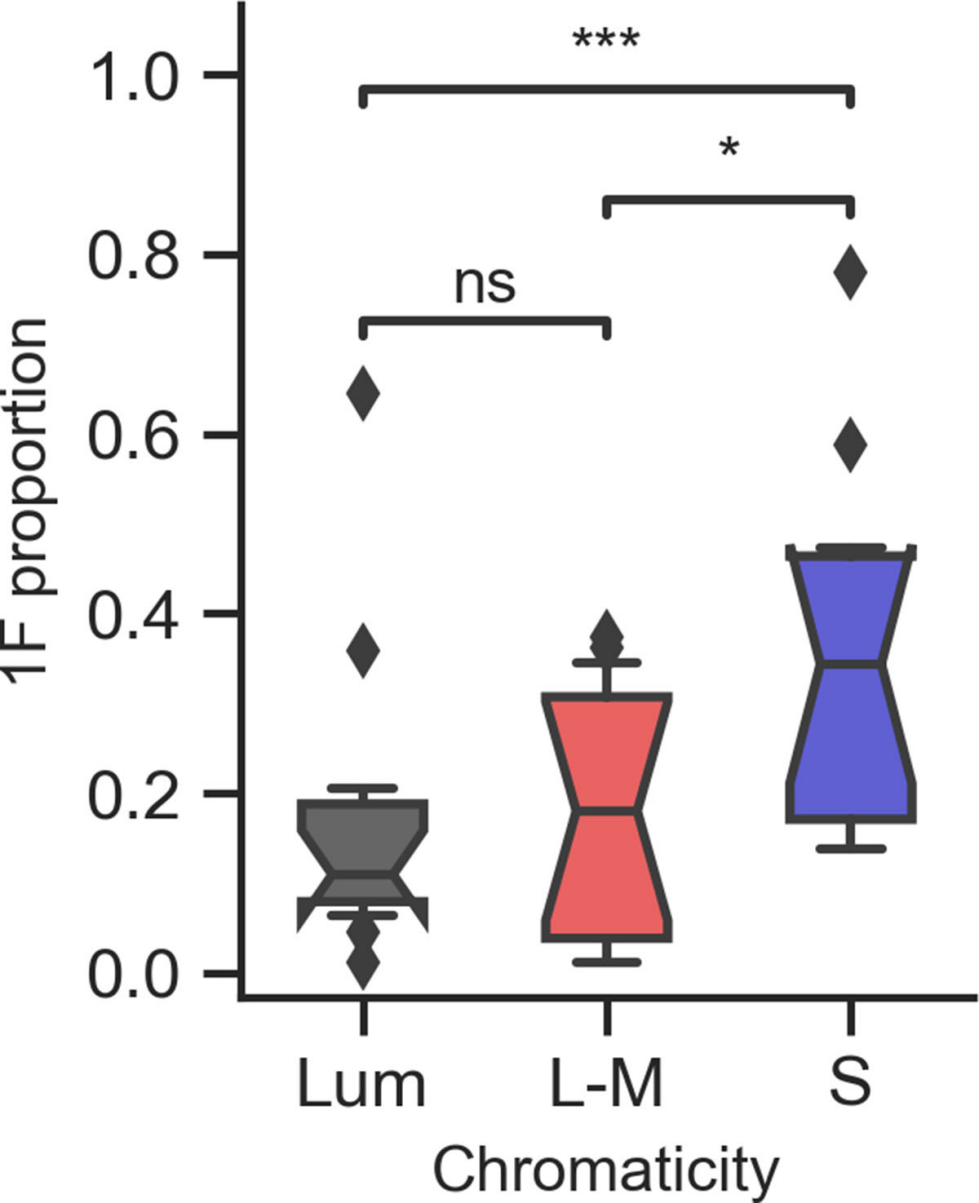
Boxplots showing the proportion of response at 1*F* terms ($\frac{1F}{1F+2F}$ ) by chromaticity for full-field (disk) conditions.

The distribution of response power between 1*F* and 2*F* can be measured by calculating a proportion of 1*F* to the total 1*F* and 2*F* response for each participant and full-field monocular condition. The 1*F* value allows us to estimate the asymmetry between simple cell populations responding to inputs of different polarity. Should there be no asymmetry, we would expect a 1*F* proportion close to 0. Figure 4 shows boxplots of the 1*F* proportions, averaged across both eyes. Although all monocular disk conditions generate some 1*F* signal, we see the greatest asymmetry in the S-cone conditions compared with both luminance and L-M, with paired-sample *t*-tests revealing significant differences between S-cone and luminance (*p* < 0.001) and S-cone and L-M (*p* = 0.026) but not between luminance and L-M conditions.

### Intermodulation terms

**Figure 5.**
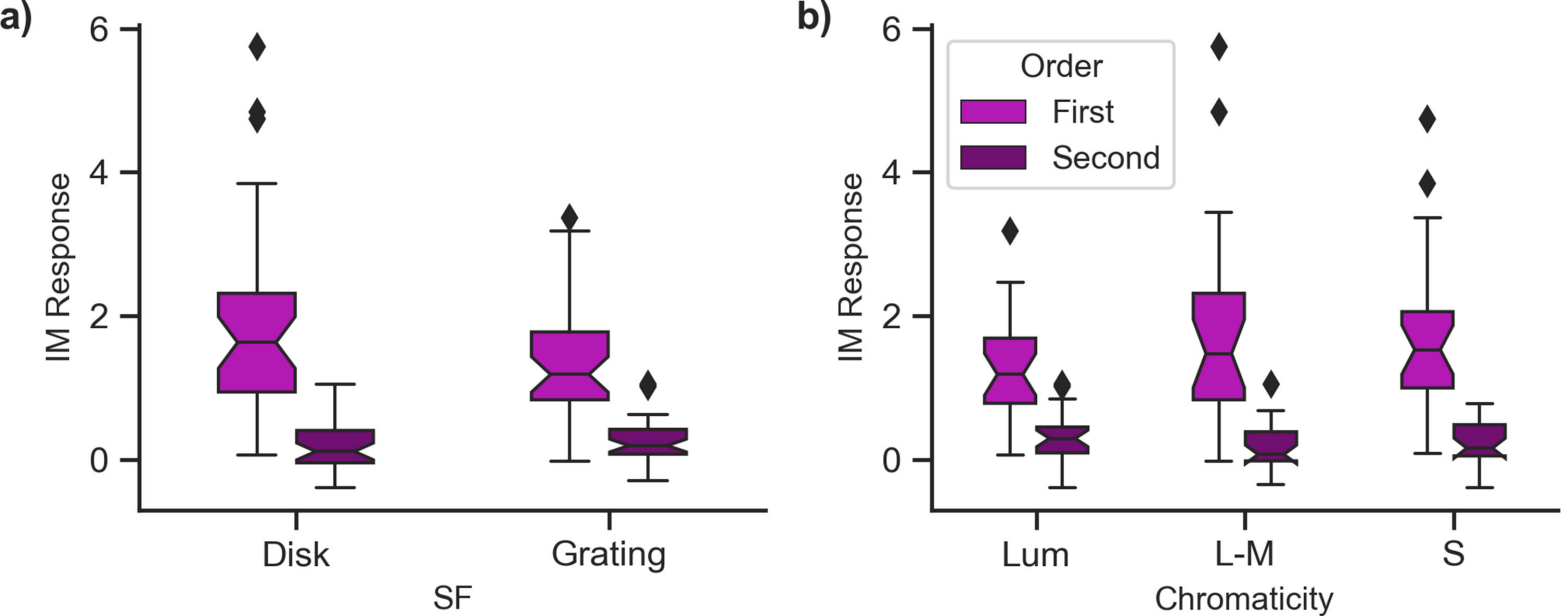
Boxplots showing the average signal above noise at combinations of the first or second harmonics (i.e., 1*F*1+1*F*2 and 1*F*2–1*F*1 or 2*F*1+2*F*2 and 2*F*2–2*F*1) by spatial frequency (**a**) and chromaticity (**b**).

Individual simple and complex cells are associated with 1*F* and 2*F* outputs, respectively. As previously discussed, EEG measures a population response, and, as simple cells responding to both polarities are present within the visual system, we see mostly responses at 2*F*. But, 1*F* signals driven by simple cells are still present within the visual system. IM occurs at combinations of the self-term responses in binocular conditions only. Figure 5 shows boxplots of the average response at first-order IM terms (i.e., 1*F*2–1*F*1 = 2 Hz and 1*F*2+1*F*1 = 12 Hz) and second-order IM terms (i.e., 2*F*2–2*F*1 = 4 Hz and 2*F*2+2*F*1 = 24 Hz). Here, we found that simple cells must be involved in binocular combinations because we observed large and significant IM responses at combinations of 1*F* compared with noise (2 Hz, *p* ≤ 0.008; 12 Hz, *p* ≤ 0.007), but not at combinations of 2*F*. This finding differs noticeably from the self-term responses, which are present at the second harmonics. As such, the binocular combinations that we measure may be driven primarily by simple cells rather than complex cells.

In summary, we measured robust stimulus–driven responses in both eyes. These responses were primarily at the second harmonic of the input frequency (2*F*), as we would expect for contrast-reversing stimuli. However, we do see some evidence of response at the first harmonic (1*F*) which may be due to asymmetries in early ON/OFF pathways or stimulus edge effects. We also measured strong binocular interactions. When the inputs were binocular, the *F*1 and *F*2 responses were significantly diminished (consistent with a binocular suppression computation) and IM terms appeared to arise largely in the simple cells responding at 1*F*. Finally, the overall strength of suppression expressed relative to the monocular responses did not appear to depend on spatial frequency or chromaticity.

### Experiment 2: control

**Figure 6.**
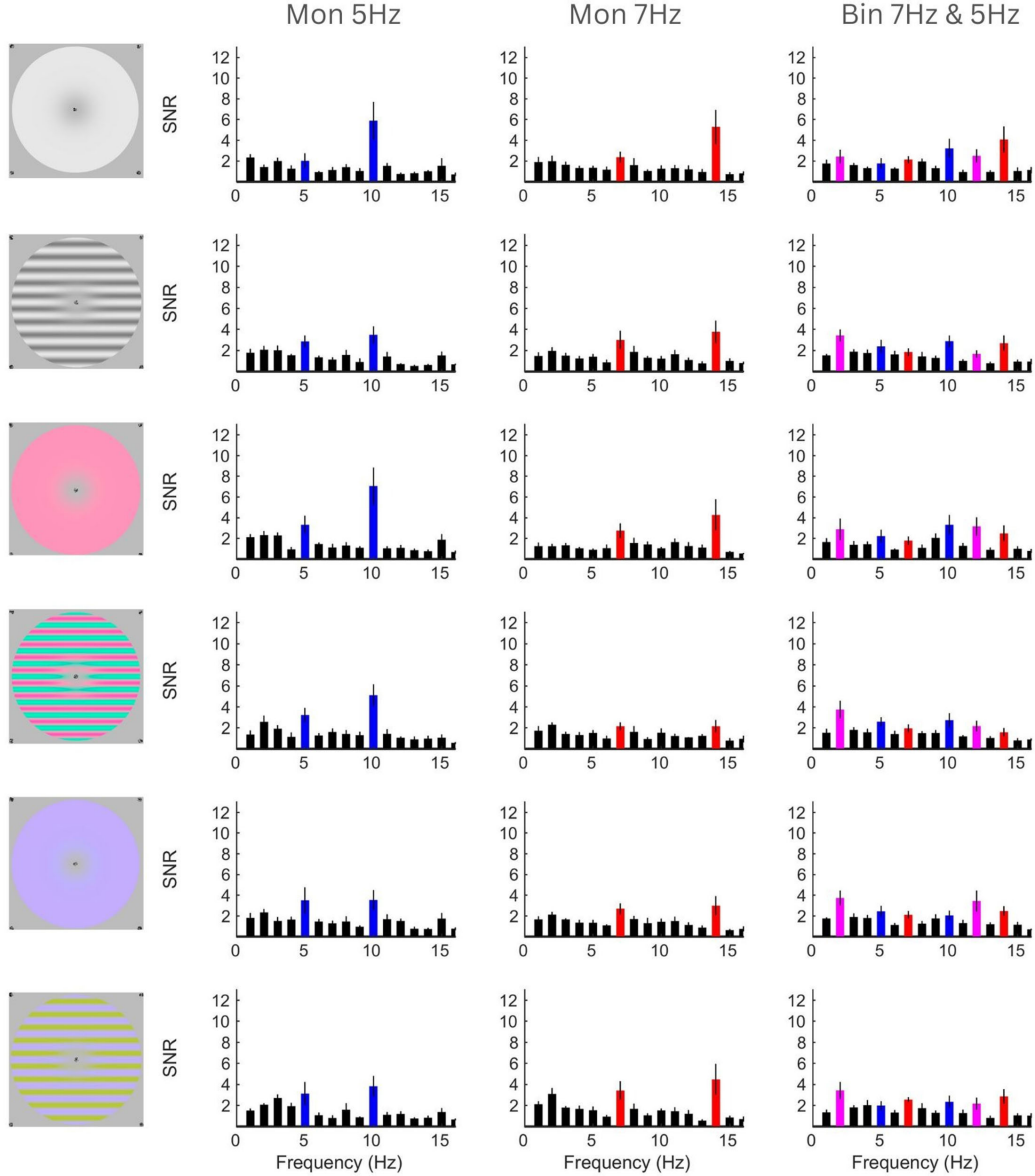
Signal-to-noise ratios of EEG control data for each condition. Columns represent left monocular 5 Hz, right monocular 7 Hz, and binocular conditions, respectively. Rows represent the different stimulus conditions. Red, blue, and magenta bars highlight responses dependent on 7-Hz input, 5-Hz input, or binocular interaction, respectively. Error bars indicate 1 *SE*.

The 5-Hz flicker generally produced a higher 2*F* response than 7 Hz. In Experiment 1, frequency and eye were linked: 5 Hz was always presented to the right eye and 7 Hz to the left eye. To ensure that the response asymmetries we observed were due to differences in temporal frequency sensitivity and not a population-level eye dominance effect, the experiment was re-run with the eye frequency tags switched. Overall, the frequency-dependent results were very similar to those in Experiment 1 (see Figures 2 and 6). This suggests that the differences in response amplitude between frequencies are likely due to a frequency preference in the visual system rather than overall ocular dominance.

## Discussion

The columnar model of Livingstone and Hubel makes strong predictions about the relationship among color, spatial frequency, and binocularity in primary visual cortex. Specifically, the location of a cell within an ocular dominance column determines the level of binocular input that it receives, and cells that respond primarily to low spatial frequency chromatic stimuli are clustered in the center of the ocular dominance columns in the cytochrome oxidase blobs. The cells in the so-called “interblobs” code for relatively high spatial frequency inputs where color identity is less important. Because the interblobs cross ocular dominance boundaries, cells in these regions might be expected to have more binocular interaction than those in the blobs. This model predicts a set of responses where binocularity is strongest for high spatial frequency, achromatic stimuli. We note that, although this model has been challenged many times, recent reports do support some aspects of it. For example, several papers (Chatterjee et al., 2021; Garg et al., 2019; Li et al., 2022) have used two-photon imaging to measure chromatic and spatial frequency tuning of a large population of neurons in macaque V1. These groups reported evidence that cells responsive to different directions in cone contrast space and spatial frequencies form distinct clusters and that those clusters tend to have distinct spatial relationships with the positions of cytochrome oxidase blobs. More generally, some form of anatomical (although not necessarily columnar) segregation has been observed for chromatic inputs to V1 for many decades (e.g., Blasdel & Lund, 1983; Chatterjee & Callaway, 2003; De Valois, Cottaris, Elfar, Mahon, & Wilson, 2000).

Although we did find significant differences in binocular interaction driven by both chromaticity and spatial frequency, our data do not support the simple model described above. Specifically, if low spatial frequency chromatic signals were processed exclusively by (monocular) neurons in the centers of ocular dominance columns, we would expect to measure little or no interocular suppression for these inputs. Our data do show robust overall suppression for all stimulus types, including significantly reduced absolute levels of signal reduction for isoluminant S-cone inputs. An ANOVA on the raw suppression amplitudes did find a significant effect of chromaticity on suppression, with S-cone stimuli showing the least overall suppression. However, when expressed as a percentage of the (unsuppressed) monocular signal, we found approximately the same level of relative interocular signal suppression across chromatic and spatial frequency conditions.

### ON/OFF asymmetry

Unstructured disk stimuli drive simple cells sensitive to opposite input polarities in alternation once per cycle. If the populations of these cells are equal and each cell contributes approximately the same amount to the EEG signal, then we would expect the response to these stimuli to consist only of even harmonics. Deviations from these assumptions (for example, differences in the numbers of simple chromatic cells or low spatial frequency tuned achromatic simple cells responding to different directions in color space, their response latencies, or their laminar location) will lead to odd harmonic responses. Asymmetries between these cell population responses can therefore be approximated by proportion of odd harmonic responses in the overall response to the stimuli (odd and even). Because the amplitudes of responses after 2*F* were relatively small, we measured this as the proportion of 1*F* to 1*F*+2*F*. We found asymmetries in all conditions, but these were particularly strong for the S-cone disks. Although phase ambiguity in our SSVEP data combined with our incoherent averaging across hemispheres and subjects means that we cannot assign a polarity to the asymmetry, these results are consistent with the fact that most short-wave sensitive cone cells connect to an S-ON bipolar, with S-OFF bipolars being relatively rare (less than 10% of the S-bipolar population) (Calkins, Tsukamoto, & Sterling, 1998; Dacey & Lee, 1994; Stockman, MacLeod, & DePriest, 1991). Our data also suggest smaller but still significant ON/OFF asymmetries in responses to luminance and L-M contrast. The literature suggests that there are small but significant asymmetries in amplitude, as well as response dynamics for both luminance and L-M inputs (Komban et al., 2014; Kremkow et al., 2014; Zemon, Gordon, & Welch, 1988). We note also that the luminance ON and OFF pathways are differentially affected by amblyopia (Pons, Jin, Mazade, Dul, Zaidi, & Alonso, 2019), suggesting that these asymmetries are, to some degree, determined environmentally.

### Normalization intermodulation terms

At the final population level, simple and complex cells both generate 2*F* responses: Individual simple cells respond at 1*F*, but the SSVEP signal averages over approximately equal populations of cells responding in antiphase with each other and the result is frequency doubled. Complex cells respond to both phases of the contrast-reversing stimuli, and the output of individual cells is therefore naturally at even harmonics. However, normalization computations can generate IM terms that reflect the different response characteristics of individual cell types. Specifically, populations of simple cells can in principle give rise to IM terms at differences of odd terms but complex cells cannot.

Most of the intermodulation power in our responses is at these first-order differences (specifically at 1*F*2–1*F*1 and 1*F*2+1*F*1) rather than at sums and differences of the even harmonics. This suggests that the majority of the binocular interactions are occurring between cells that would typically be classed as simple, or single-opponent.

This dominance of first-order intermodulation terms may derive from the relative sizes of the underlying cell populations: Early measures found a ratio of simple to complex cells of about 3:1 in cat visual cortex (e.g., Hubel & Wiesel, 1962), but more recent estimates in primates found that the populations may be more similar (Skottun, De Valois, Grosof, Movshon, Albrecht, & Bonds, 1991) and, importantly, that the strict dichotomy between simple and complex cells might, at least in part, be an artifact of the analysis method and that the cell types might exist on a continuum (Mechler & Ringach, 2002). It is also possible that higher order IM terms may be generated in cells that do not make strong contributions to the farfield visually evoked potential because of the fine details of cellular physiology and geometry. One aspect of our data is clear, however: The presence of significant IM terms at sums and differences of the 1*F*1 and 1*F*2 inputs unequivocally demonstrated that binocular interactions were occurring between neurons that were responding at the first harmonic of the input stimulus—in other words, cells that would, by most definitions, be termed simple or single-opponent cells. This interaction was present even in cells responding to low spatial frequency chromatic inputs. Previous single-unit work with achromatic stimuli has identified binocularity in both simple and complex cells (Anzai, Ohzawa, & Freeman, 1999a; Anzai, Ohzawa, & Freeman, 1999b; Cumming & DeAngelis, 2001; Ohzawa & Freeman, 1986). Although not all binocular interactions necessarily lead to stereoscopic depth computations, our data indicate that chromatically tuned cells could support stereopsis, and recent data from our group have shown that stimuli constrained to isoluminant color directions can generate robust percepts of motion in depth based solely on changing disparity signals (Kaestner, Maloney, Wailes-Newson, Bloj, Harris, Morland, & Wade, 2019).

### Binocular color

Binocular interaction can be measured by the amount of self-term suppression and increase in IM terms in binocular conditions relative to monocular conditions. In all conditions, we saw a significant decrease in self-term responses and a significant increase at IM terms, indicating that all chromatic channels showed interocular suppression. The original Livingstone and Hubel model makes a strong prediction: Binocular interactions for low spatial frequency color (and particularly S-cone stimuli) should be weaker than binocular interactions for high spatial frequency luminance contrast. In many primates, cytochrome oxidase blobs lie at the centers of the ocular dominance columns and therefore tend to be monocular in their input preferences. Chromatic sensitivity is often thought to be highest, and spatial frequency sensitivity lowest, in these blob regions. Our data do not support this strict segregation of chromaticity, binocularity, and spatial frequency tuning.

One point to consider is that signals in primary visual cortex pass through more than one synapse. Although processing might be restricted initially to restricted anatomical regions, neurons in later stages may pool from wider spatial extents, integrating information about multiple features (including eye of origin) and reducing the effect of the initial pathway segregations (Sincich & Horton, 2005). Our recording technique provides a population average from all responses in V1 and also includes small contributions from neighboring visual areas (Poncet & Ales, 2023), thus we are unable to dissect out the contributions of the different stages of V1 computations.

Individual differences have been found in binocular interaction (Baker & Graf, 2009). Intriguingly, ocular dominance columns are both highly variable across individuals in some primate species and are also not required for stereoscopic vision (Horton & Adams, 2005). It is therefore possible that, in some of our subjects, chromatic inputs may be restricted to cytochrome oxidase blobs, but these blobs may not be strictly segregated into purely monocular regions.

## Conclusions

Here, we have shown that SSVEP responses can be used to examine the computations governing chromatic and achromatic signal transduction and binocular signal combination. We observed that parameters that govern these computations, including the amount of binocular normalization, the ratio of simple to complex cells, and the ratio of OFF to ON cell responses, can be constrained by the properties of the SSVEP response spectrum. Our results are particularly relevant to “standard” models of how anatomy might constrain the processing of chromatic and achromatic inputs in early visual cortex—particularly the prediction that binocular integration of low spatial frequency chromatic stimuli might be relatively weak because they are processed in the centers of ocular dominance columns. We found little evidence for this, although there was some evidence of weaker binocular interactions for S-cone inputs. If anatomical factors such as blobs and ocular dominance domains are correlated with early tuning for chromaticity or spatial frequency, our data suggest that they do not act as tight constraints on subsequent computations that combine information across eyes. Finally, in this study, we restricted our dichoptic stimuli to the same chromatic axis and spatial frequency: Both eyes saw the same stimuli with different temporal frequency tags. It is also possible to use similar analysis logic to examine the way in which different chromatic channels are combined in visual neurons (Watts, Rozman, Somers, Gunel, Racey, Barnes, & Bosten, 2023), and it would be a natural extension of this technique to ask how this cross-channel integration depends on ocularity.
